# Abscisic acid induced a negative geotropic response in dark-incubated *Chlamydomonas reinhardtii*

**DOI:** 10.1038/s41598-019-48632-0

**Published:** 2019-08-19

**Authors:** Layla Al-Hijab, Adam Gregg, Rhiannon Davies, Heather Macdonald, Michael Ladomery, Ian Wilson

**Affiliations:** University of the West of England, Bristol; Department of Applied Sciences, Faculty of Health and Applied Sciences; Frenchay Campus, Coldharbour Lane, Bristol, BS16 1QY United Kingdom

**Keywords:** Microbiology, Tropism

## Abstract

The phytohormone abscisic acid (ABA) plays a role in stresses that alter plant water status and may also regulate root gravitropism and hydrotropism. ABA also exists in the aquatic algal progenitors of land plants, but other than its involvement in stress responses, its physiological role in these microorganisms remains elusive. We show that exogenous ABA significantly altered the HCO_3_^−^ uptake of *Chamydomonas reinhardtii* in a light-intensity-dependent manner. In high light ABA enhanced HCO_3_^−^ uptake, while under low light uptake was diminished. In the dark, ABA induced a negative geotropic movement of the algae to an extent dependent on the time of sampling during the light/dark cycle. The algae also showed a differential, light-dependent directional taxis response to a fixed ABA source, moving horizontally towards the source in the light and away in the dark. We conclude that light and ABA signal competitively in order for algae to position themselves in the water column to minimise photo-oxidative stress and optimise photosynthetic efficiency. We suggest that the development of this response mechanism in motile algae may have been an important step in the evolution of terrestrial plants and that its retention therein strongly implicates ABA in the regulation of their relevant tropisms.

## Introduction

Photosynthetic green algae evolved 1 to 1.5 billion years ago when a eukaryotic heterotroph encapsulated a cyanobacterium which ultimately formed a plastid^1^. Numerous lineages diverged^2^, including the chlorophytes and charophytes, the progenitors of terrestrial plants which appeared ≈500 million years ago^3^. The aquatic to terrestrial transition must have posed a significant challenge to algae and environmental differences required the adaptive evolution of protective mechanisms enabling them to become high-light and desiccation tolerant and sessile at the water surface. It is likely that such mechanisms derived from those which had already evolved to ensure their survival. For example, Chlamydomonas sp. alter their depth depending on light level and quality to attenuate photo-oxidative stress^4^. As such mechanisms became more effective, the depth to which algae needed to descend presumably reduced, enabling them to thrive closer into the shoreline.

Algal genomes encode rudimentary synthesis and signalling pathways for the majority of phytohormones, including ABA^5^. In terrestrial plants ABA plays a role in seed dormancy and germination, stomatal movements and in other responses to numerous stresses that alter their water status^6^. ABA may also negatively regulate root gravitropism^7^ and root hydrotropism^8^, but the underlying signalling mechanisms remain unclear. However, its algal role remains poorly defined with only a few papers suggesting its involvement in stress responses such as those resulting from desiccation^9^ and salinity^10^ and in the initiation of the cell cycle^11,12^. ABA treatment has also been shown to increase lipid accumulation in Chlorella^13^.

Within the complexity of multicellular, multi-organ, terrestrial plants it has proved difficult experimentally to isolate the many potential roles of ABA away from its major stress-related role in preventing water loss. ABA loss of function mutants exhibit pleiotropic phenotypes and simply applying ABA to plants induces an immediate stress response. Thus, properly defining a role for ABA in a photosynthetic progenitor of land plants where water stress is not normally an issue, the green algae may give valuable insight into its other roles. Therefore, the aim of this study was to define a specific role for ABA in the motile green alga, *Chlamydomonas reinhardtii*, providing evidence that this phytohormone mediates the light-dependent diurnal rhythm of up and down gravitaxis of the algae in the water column.

## Results

### ABA altered the photosynthetic efficiency of *C. reinhardtii* CC-1021

**Figure 1:**
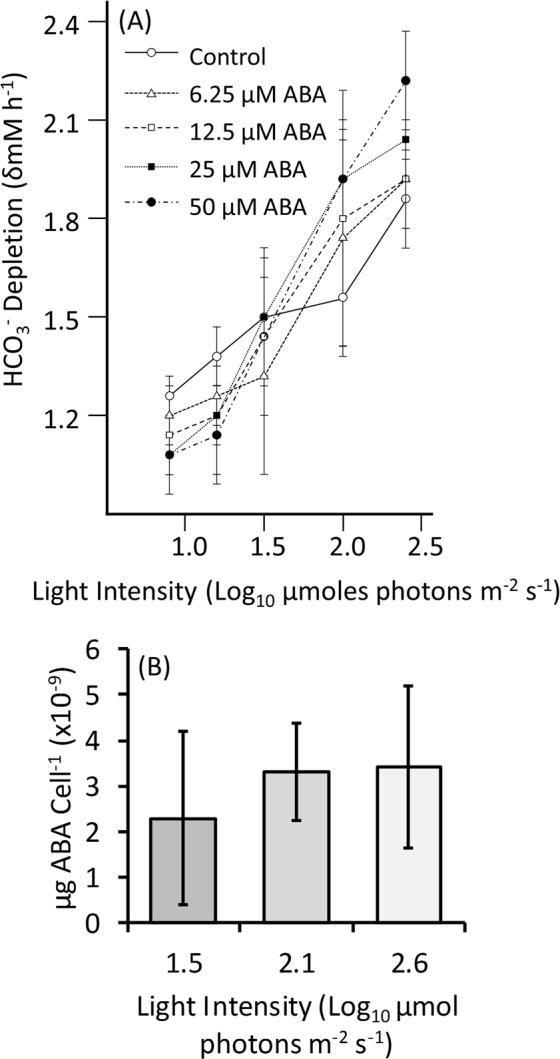
Exogenous abscisic (ABA) acid differentially altered HCO_3_^−^ uptake by *C. reinhardtii* in a light-intensity-dependent manner. Mid log phase (A_750_ = 0.3) algal cultures were immobilised in alginate gel beads containing the concentrations of ABA indicated and in (**A**) were incubated for 1 h under different light intensities (7.6 to 223.2 µmoles photons m^−2^ s^−1^) with bicarbonate indicator buffer in TAP media. Depletion of HCO_3_^−^ in the media was monitored as the change in absorbance in the indicator buffer at 550 nm after the incubation period with conversion to the change in the concentration of HCO_3_^−^ in the TAP media by reference to a standard calibration. Shown are the mean δHCO_3_^−^ (mM) h^−1^ of n = 5 replicates with errors as the 95% confidence intervals around the means in each case. In (**B**) mid log phase (A_750_ = 0.3) algal cultures were sampled 1 h into the photoperiod and subsequently exposed for 1 h to the different light intensities indicated. Algal cells were pelleted from n = 3 replicate 25 mL aliquots of culture. Pellets were extracted and assessed for ABA content by competitive ELISA (MyBioSource Inc.). Data are shown as the mean ABA content cell^−1^ with error bars shown as +/− the 95% confidence interval around the mean in each case.

Reasoning that high-light-induced photo-oxidation likely poses the main threat to photosynthetic algae, here the initial investigation focused on the effect of ABA on the photosynthetic efficiency of *C. reinhardtii* by measuring the ability of treated algae to deplete their growing media of dissolved HCO_3_^−^ under different light levels. Alginate-bead-encapsulated *C. reinhardtii* with different concentrations of ABA were incubated under different light levels in media containing bicarbonate indicator. Under relatively high-light illumination (223.2 µmoles photons m^−2^ s^−1^), 50 µM ABA significantly (P < 0.05 by Tukey Kramer’s test) increased mean HCO_3_^−^ depletion of the media by the algae by ≈20% compared to the control algae under the same light levels. Conversely, at low-light (7.59 µmoles photons m^−2^ s^−1^) 50 µM ABA significantly reduced the mean HCO_3_^−^ depletion of the media by the algae by ≈14.5% (Fig. 1A). Lower ABA doses had an intermediate effect. At 32 µmoles photons m^−2^ s^−1^ ABA had no significant effect on the mean HCO_3_^−^ depletion of the media by the algae regardless of the dose used. When grown at 45 µmoles photons m^−2^ s^−1^ and sampled 1 h into the photoperiod, subsequent exposure of the algae to high light conditions at 375 µmoles photons m^−2^ s^−1^ increased their mean endogenous ABA content by 1.4 fold (Fig. 1B), but not in a statistically significant manner.

### ABA and light signal together to position *C. reinhardtii* CC-1021 vertically in the water column

**Figure 2:**
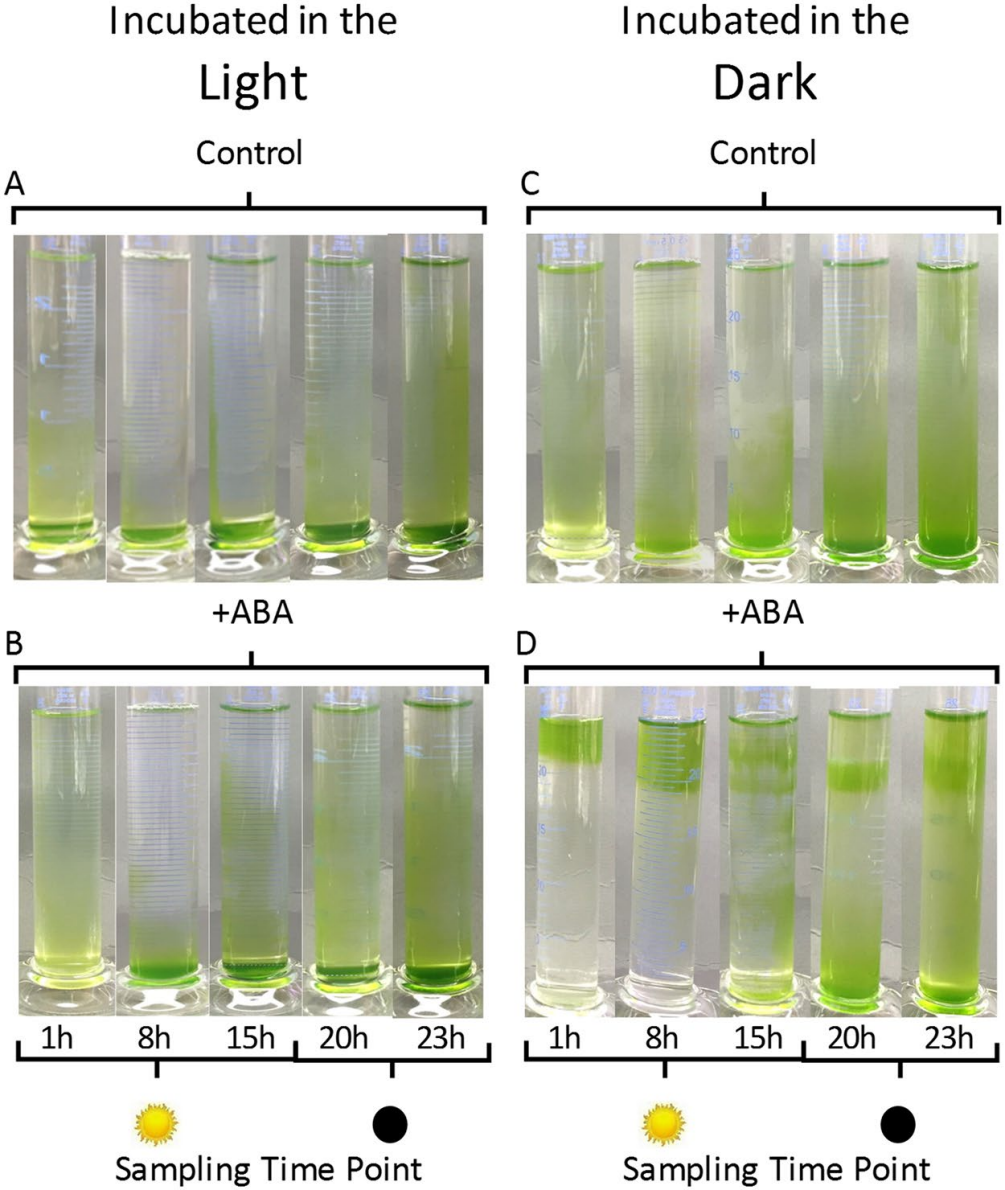
Exogenous abscisic acid (ABA) induced a negative geotropic movement response in *C. reinhardtii* CC-1021. Algal cultures were grown under a 16 h photoperiod to the mid log phase (A_750_ = 0.3) growth stage and were sampled at the time points indicated over a 24 h period where the 16 h photoperiod commenced at time = 0 h. Fully dispersed algal samples were placed in measuring cylinders +/−50 µM ABA to form a 10 cm vertical water column and for 50 min were either illuminated from above with high-light (319.8 µmoles photons m^−2^ s^−1^) (**A**,**B**) or placed in the dark (**C**,**D**). Shown is a representative image of n = 5 replicate experiments indicating the positions attained by the algae immediately following the period of incubation in either the light or dark.

**Figure 3:**
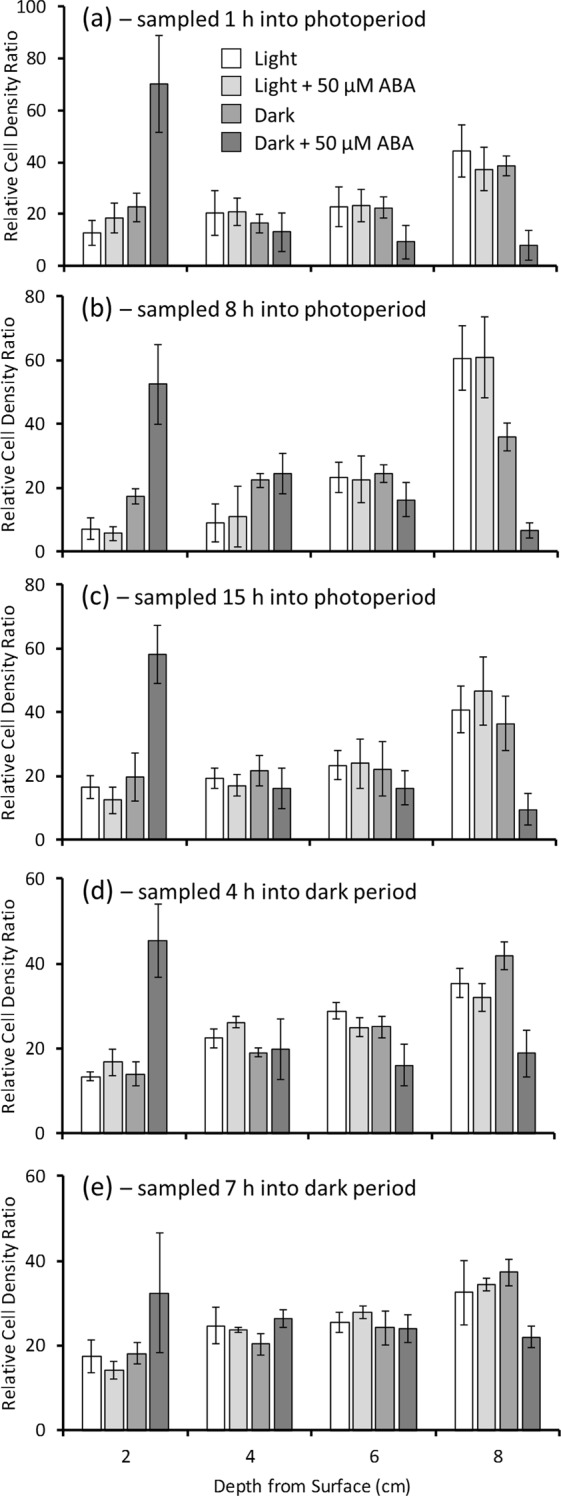
Exogenous abscisic acid (ABA) induced a negative geotropic movement response in *C. reinhardtii* CC-1021. Algal cultures were grown under a 16 h photoperiod to the mid log phase (A_750_ = 0.3) growth stage and were sampled at (**a**) 1 h, (**b**) 8 h, (**c**) 15 h, (**d**) 20 h and (**e**) 23 h during a 24 h period where the 16 h photoperiod commenced at time = 0 h. Fully dispersed algal samples were placed in measuring cylinders +/−50 µM ABA to form 10 cm vertical culture columns and for 50 min were either illuminated from above with high-light (319.8 µmoles photons m^−2^ s^−1^) or placed in the dark. After incubation the A_750_ at the depths indicated was measured and the relative cell density ratios at each depth were determined within each treatment. Shown are the mean data of n = 5 replicate experiments +/− the 95% confidence intervals around the mean in each case.

Since Chlamydomonas are known to respond to changing light levels by exhibiting either positive or negative phototaxis with subsequent adaptation^4,14^ and since ABA treatment differentially altered the photosynthetic efficiency of the algae in a light dependent manner, thus indicating an ABA/light signalling interaction, it was reasoned that ABA signalling could be involved in determining their vertical position in the water column. *C. reinhardtii* were thus sampled at different time points over a 24 h cycle and fully dispersed samples incubated in upright measuring cylinders +/−50 µM ABA under either overhead, high-light illumination (319.8 µmoles photons m^−2^ s^−1^) or in the dark for 50 min (Figs. 2 and 3).

Control algae with overhead illumination retreated downwards to an extent dependent on their sampling point in the 24 h light/dark cycle (Figs. 2A and 3). Those sampled mid photoperiod retreated significantly (P < 0.05 by χ^2^ test of goodness of fit) further downwards than those sampled earlier and towards the end of the photoperiod. Those sampled in the dark period and then illuminated from above retreated downwards less than those sampled at any time point in the photoperiod, with those sampled at the end of the dark period tending to remain more dispersed when returned to the light, but not to a significant extent (P > 0.05 by χ^2^ test of independence). ABA treatment marginally reduced this negative phototactic response to high-light at some time points, but not at others (Figs 2B and 3). Algae sampled 1 h into the photoperiod and light-incubated with 50 µM ABA remained more dispersed than the controls, but not to a statistically significant extent. When sampled in the middle of the photoperiod ABA had no significant effect, while algae sampled at the end of the photoperiod and in the middle of the dark period showed reduced downward movement when treated with ABA, but again, not to a significant extent. There was no observed difference in the position attained by the algae sampled at the end of the dark period when placed in the light regardless of ABA treatment.

Algae of control samples, taken during the photoperiod and placed in the dark, moved downwards to an extent depending on the duration of illumination already received (Fig. 2C). Algae sampled after 1 h of the photoperiod remained dispersed during their subsequent dark incubation, while those sampled at 8 and 15 h into the photoperiod showed a progressive downward movement. Algae sampled during the dark period at 20 h into the 24 h cycle and maintained in the dark moved towards the bottom, while those sampled at 23 h were more dispersed. However, overall the distribution of the algae when incubated in the dark was not significantly (P > 0.05 by χ^2^ tests of independence) different regardless of the sampling time point. Intriguingly, when samples were treated with ABA and placed in the dark the algae moved upwards significantly (P < 0.05 by χ^2^ tests of goodness of fit) to form discrete bands near to the surface (Fig. 2D). The depth at which the ABA-treated algae banded depended on their sampling time over the 24 h cycle. Those sampled after the first hour of the photoperiod banded at 0 to 2 cm from the surface. Those sampled later into the photoperiod still exhibited a significant negative geotropism, but banded at two increased depths. Cells sampled towards the end of the photoperiod and into the dark period were less responsive to ABA treatment in the dark, although a proportion of the cells still moved upwards to form a discrete band 2 to 4 cm from the surface.

**Figure 4:**
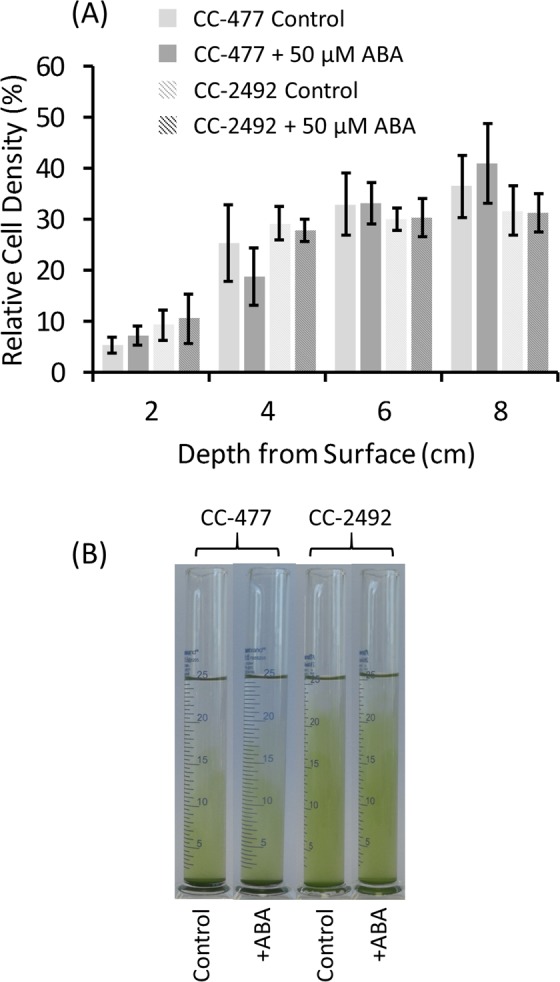
In the absence of light exogenous abscisic acid (ABA) failed to induce a negative geotropic movement in the *C. reinhardtii* mutant strains CC-477 and CC-2492 that lack functional flagella. Algal cultures of the mutant strains were grown under a 16 h photoperiod to the mid log phase (A_750_ = 0.3) growth stage and were sampled 1 h into the photoperiod. Fully dispersed algal samples were placed in measuring cylinders +/−50 µM ABA to form 10 cm vertical culture columns and for 50 min were incubated in the dark. After incubation the A_750_ at the depths indicated was measured and the relative cell density ratios at each depth were determined within each treatment. Shown in (**A**) are the means of data from n = 4 replicate experiments for each mutant +/− the 95% confidence intervals around the means in each case. Shown in (**B**) is a representative image of the positions attained by the algae immediately following the period of incubation.

To determine that the effect of ABA in the dark-incubated algae resulted from active swimming and not from altered buoyancy two independent mutants, CC-477 (*BLD1* gene mutation that lacks functional intra-flagellar transport protein 52 and is unable to assemble flagella) and CC-2492 (loss of function mutation of the flagellar outer dynein arm light chain 2 (*DLC2*) gene) of *C. reinhardtii* that lacked functional flagella were sampled at the time point, 1 h into the photoperiod, when the CC-1021 strain of the algae had appeared most ABA responsive and were similarly incubated +/−50 µM ABA in the dark (Fig. 4). In the absence of ABA the dark-incubated algal cells of either mutant settled downwards such that the A_750_ at increasing depths were significantly (P < 0.05 by Tukey Kramer’s test) higher than at shallower depths. The addition of 50 µM ABA in the dark had no significant (P > 0.05 by χ^2^ tests of independence) effect on this distribution of the algal cells of either mutant suggesting that the presence of functional flagella was required for the ABA-induced upwards movement in the dark observed with the wild type *C. reinhardtii* strain CC-1021.

### NAA, H_2_O_2_ and ACC did not affect the vertical positioning of *C. reinhardtii* CC-1021 in either the light or dark

**Figure 5:**
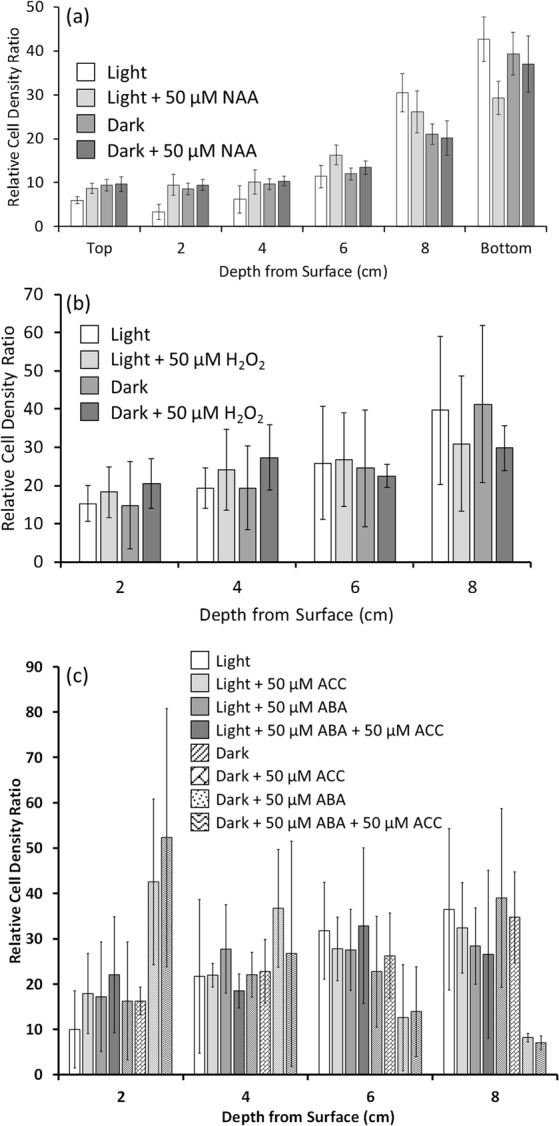
Exogenous 1-naphthaleneacetic acid (NAA), 1-aminocyclopropane-1-carboxylic acid (ACC) and hydrogen peroxide (H_2_O_2_) did not significantly affect the vertical positioning of *C. reinhardtii* CC-1021 in the dark. Algal cultures were grown under a 16 h photoperiod to the mid log phase (A_750_ = 0.3) growth stage and were sampled 8 h into the 16 h photoperiod. Fully dispersed algal samples were placed in measuring cylinders +/− either 50 µM (**a**) NAA, (**b**) H_2_O_2_ or (**c**) ACC, ABA and ACC + ABA to form 10 cm vertical culture columns and for 50 min were either illuminated from above with high-light (319.8 µmoles photons m^−2^ s^−1^) or placed in the dark. After incubation the A_750_ at the depths indicated were measured and the relative cell density ratios for each depth were determined within each treatment. Shown are the mean data of either n = 5 (**a**) or n = 3 (**b**,**c**) replicate experiments +/− the 95% confidence intervals around the means in each case.

Since the ABA-induced upward movement of the algae in complete darkness suggested that the signalling of this phytohormone may have been acting gravitropically, it was deemed prudent to ascertain whether the main gravitropic phytohormone, auxin, had a similar effect on Chlamydomonas. Thus, mid log phase growth algae were sampled mid photoperiod, treated with the cell-permeable, synthetic auxin 1-naphthaleneacetic acid (NAA) and were incubated as for the ABA experiments above (Fig. 5a). Compared to the untreated control algae, NAA at 50 µM had no significant (P > 0.05 by χ^2^ test of independence) effect on the vertical positioning of the algae in the water column when they were incubated in either the light or the dark. When the algae were incubated in the light there was some indication that NAA had a small effect and although the algae still generally retreated downwards away from the high-light, the proportion remaining at shallower depths was increased with the auxin treatment, but overall the distribution of the algae with and without NAA was not statistically significantly different.

In ABA responses such as those which occur in plant guard cells that affect stomatal movements, downstream hydrogen peroxide synthesis and signalling has been shown to be required. Here, when *C. reinhardtii* were sampled at mid photoperiod, treated with 50 µM H_2_O_2_ and incubated as for the ABA experiments above there was no significant (P > 0.05 by χ^2^ test of independence) effect on the vertical positioning of the algae in the water column when they were incubated in either the light or the dark (Fig. 5b).

Ethylene is antagonistic to a number of plant ABA responses. Here, algae were sampled mid photoperiod and treated with either the precursor of ethylene synthesis, 1-aminocyclopropane-1-carboxylic acid (ACC), either alone or with ACC and ABA together and were incubated as for the ABA experiments above. In either case, ACC treatment had no significant (P > 0.05 by χ^2^ test of independence) effect on the vertical positioning of the algae in the water column when they were incubated in either the light or the dark and no significant (P > 0.05 by χ^2^ test of independence) effect on the distribution of the algae induced by ABA (Fig. 5c).

### *C. reinhardtii* CC-1021 exhibited differential, light-dependent taxis to a fixed source of exogenous ABA

**Figure 6:**
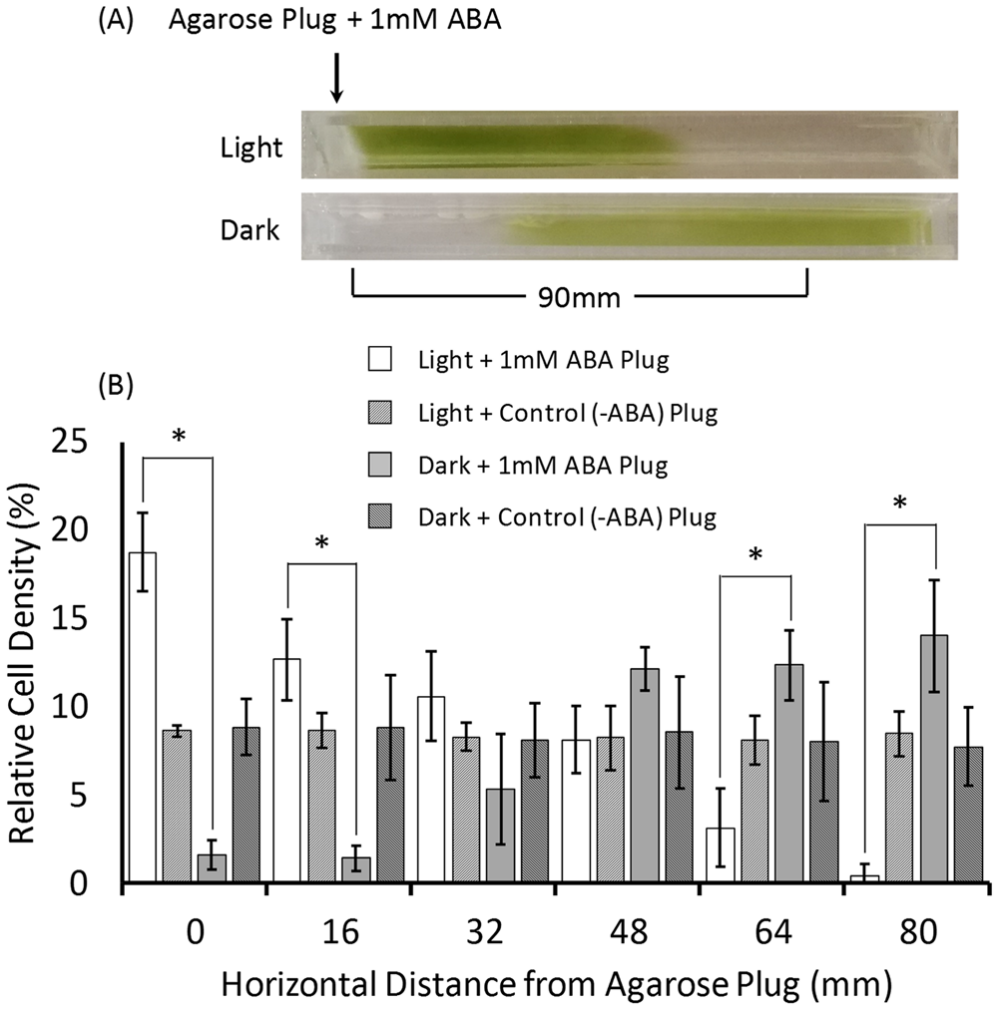
*C. reinhardtii* CC1021 showed opposite taxis to a fixed source of exogenous abscisic acid (ABA) in the light and dark. Algal cultures were grown under a 16 h photoperiod to the mid log phase (A_750_ = 0.3) growth stage and were sampled mid photoperiod. Fully dispersed samples of the algal culture were placed in open top 1.5 × 1.5 × 12 cm light-permeable acrylic troughs with a plug of agarose containing 1 mM ABA at the end indicated and were immediately placed horizontally either in the light (45 µmoles photons m^−2^ s^−1^) or the dark for 50 min. After incubation samples were simultaneously taken at incremental distances from the agar plug and their A_750_ measured. Shown in (**A**) is a representative image indicating the positions attained by the algae immediately following the incubation period. The treatment-dependent relative cell densities at increasing distances from the agar plugs is shown in (**B**). Significant (P < 0.05 by χ^2^ tests of goodness of fit) differences in the proportion of algal cells found at various distances from the ABA-containing agar plug between the light and dark incubations of the algae are indicated^(*)^.

In the dark, ABA induced the algae to abandon their usual run and tumble manner of locomotion^15^ and to swim upwards. The question was, how did cells employ the ABA signal to do this? It was reasoned that the directional response of algae to the ABA signal could be light reversible. Thus, light-permeable acrylic troughs in which had been placed at one end an agarose gel plug containing 1 mM ABA were filled with fully dispersed suspensions of algae and then immediately placed horizontally either with surrounding illumination or in the dark for 1 h. In the light algae moved towards the ABA gel plug, while conversely, in the dark they swam away (Fig. 6). The use of lower concentrations (0.5 and 0.25 mM) of ABA in the gel plug resulted in a similar, but intermediate response (data not shown).

### Endogenous ABA levels were diurnally altered in *C. reinhardtii* CC-1021

**Figure 7:**
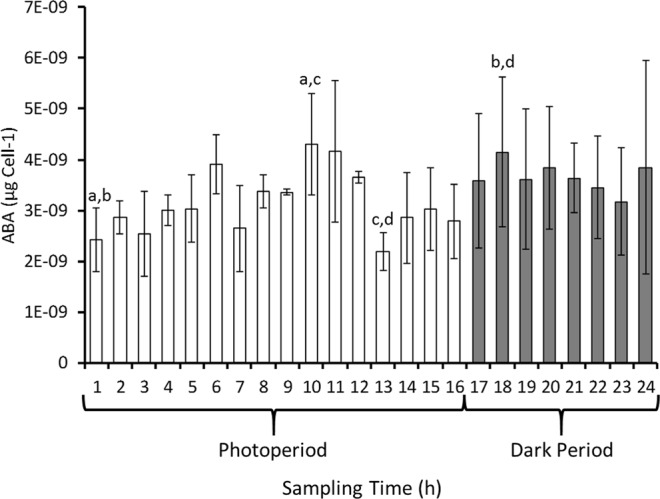
Endogenous abscisic acid (ABA) levels in *C. reinhardtii* CC-1021. Algal cultures were grown under a 16 h photoperiod to the mid log phase (A_750_ = 0.3) growth stage and were sampled at the time points indicated over a subsequent 24 h period where the 16 h photoperiod commenced at time = 0 h. Algal cells were pelleted from n = 3 replicate 25 mL aliquots of culture. At each sampling point the A_750_ of the cultures were determined to assess cell numbers. Pellets were extracted and assessed for ABA content by competitive ELISA (MyBioSource Inc.). Data are shown as the mean ABA content cell^−1^ with error bars shown as +/− the 95% confidence interval around the mean in each case. Selected significant (P < 0.05 by 2 sample t-test) pairwise differences in mean ABA levels are indicated^(a to d)^.

Overall, the results suggest that in the algae ABA signalled for an upward movement, mediated by light levels and potentially the circadian rhythm. To understand the possible contribution of circadian regulation, endogenous ABA levels of the algae were measured over the 24 h cycle (Fig. 7). Mean ABA levels varied between 2.2 × 10^−9^ to 4.3 × 10^−9^ µg cell^−1^, which assuming a mean *Chlamydomonas reinhardtii* cell volume^16^ of 270 µm3 corresponds to an endogenous concentration range of 30.8 to 60.3 nM. During the first 10 h of the photoperiod ABA levels increased by 1.8x to a maximum then declined significantly (P < 0.05 by 2 sample t-test) to a minimum at 13 h. Thereafter, ABA levels significantly (P < 0.05 by 2 sample t-test) increased again reaching near their maximum 2 h into the dark period, then remained stable until the end of the 24 h period. The time at which the level of ABA was lowest was that at which they began to form multiple bands when placed in the dark with ABA.

## Discussion

ABA is involved in plant responses to high-light^17^, which reduces photo-oxidative damage to PSII, and has been shown to increase algal mass under stress^18^. The results presented here suggest that a high-light and ABA signalling interaction increased algal photosynthetic efficiency in terms of HCO_3_^−^ uptake. Why ABA reduced algal HCO_3_^−^ uptake under low light is unclear, but perhaps, as in terrestrial plants^19^, it initiated PSII repair mechanisms under these conditions which then reduced overall photosynthetic efficiency. Previous studies have suggested that ABA may protect Chlamydomonas against photoinhibition^20^ and the results presented here would be consistent with such findings. What is interesting is the differential photosynthetic response to ABA under different light levels and the fact that, at particular light intensities, ABA had no significant effect. This suggests that ABA signalling and downstream responses in Chlamydomonas may be modulated by light and it may prove interesting to reassess this response under different light wavelengths.

It was clear that ABA and light signalled competitively to position the algae at a depth that was likely optimal for photosynthesis and that, in high-light, ABA treatment provided some protection. The lack of ABA response in the dark in the two independent flagella mutants would suggest that the ABA-induced upward movement observed in the wild type strain under similar conditions resulted from active swimming and not from a general alteration of cell buoyancy. Why ABA-treated, dark-incubated algae banded at multiple depths in specific samples is unclear, but it may indicate a difference in the sensitivity of the immature and adult zoospores to either light or ABA. The dark-incubated, ABA-treated algae banded at a lower depth when sampled 7 h into the dark period than when sampled at 1 h into the photoperiod, which suggests the influence of a circadian rhythm on the ABA response of the algae. In higher plants, many ABA-mediated responses appear to be regulated in such a circadian fashion^21^. Here, the endogenous concentrations of ABA detected in the algae were similar to those reported in other studies^22,23^. However, albeit statistically significant, the maximum 1.8 fold change in endogenous ABA levels of the algae over a 24 h period were not dramatic and considerably less than that which occurs in plants following stress treatments^24^. Here, exposure of the algae to a short period of high light intensity also only resulted in a marginal increase in ABA levels. Thus, it may be that the effect of light signalling persisted into the dark period such that the algae were only fully ABA-responsive with regard to their upward movement when biochemically prepared for exposure to the light of the early photoperiod, *i.e*. just before dawn. Additionally, as in plants^25^, it may be that ABA compartmentalisation, transport and redistribution are an important facet of its signalling capability in Chlamydomonas. The alga does possess potential homologues of genes encoding such plant ABA transporters as Arabidopsis AtABCG40 (PDR12)^26^, AtABCG25^27^ and AIT1^28^, but with only partial Chlamydomonas sequences available and varying degrees of sequence similarity (tBLASTn: AtABCG25 *vs*. XM_001690681 - 38.9% identity over 78% of query sequence; PDR12 *vs*. XM_001697838 - 39.8% identity over 95% of query sequence; AIT *vs*. XM_001692259 - 26.2% identity over 37% of query sequence) it will be important to assess whether the encoded potential protein homologues have similar functions.

The differential, light-dependent taxis response of the algae to a fixed ABA source suggested that the orientation of the algae in response to the ABA signal and their resulting direction of movement depended on the presence or absence of light. Chlamydomonas use rhodopsin-initiated Ca^2+^ currents to regulate flagella movements and thus, their orientation to light and phototactic responses^29^. Ca^2+^ signalling is also known to regulate the gravitaxis of the alga Euglena^30^. In higher plants many ABA responses similarly involve Ca^2+^ signalling^31^. For example, the opening and closing of fully-hydrated *Arabidopsis* stomata in the day/night, light/dark cycle is an ABA-regulated process^32^ involving downstream Ca^2+^ movement and signalling^31^. It will be interesting to see if the case is similar in motile algae such as Chlamydomonas.

The observation that Chlamydomonas cells move towards ABA in the light and away in the dark, taken together with the ABA-induced, negative geotropic response in the dark, strongly indicates that ABA plays a role in the gravitropic/gravitaxis response of this alga that is modulated by exposure to light. How the alga perceives ABA is then a major question as there are no orthologues of the genes encoding the plant cytosol/nuclear localised PYR/PYL/RCAR receptor family^33^ in the Chlamydomonas genome^5^. Potential orthologues of genes encoding the G-protein coupled receptor (GPCR) protein, *GCR2*^34^ and GPCR-types, *GTG1* and *GTG2*^35^, can be identified in the genome of this alga. However, it is still unclear whether or not, as has been proposed, these genes encode proteins that act as ABA receptors^36–38^. Thus, it will be important to determine the mechanisms by which algae such as Chlamydomonas perceive ABA and mechanistically how the response to its perception interacts with light signalling to determine both their orientation and direction of movement.

This study was unable to provide any evidence that either NAA, ethylene or hydrogen peroxide had any substantial effect on the vertical positioning of Chlamydomonas in the water column in either the light or dark. Green algae have been shown to contain and metabolise auxins^39^ and exogenous applications of indoleacetic acid (IAA) and NAA have been shown to improve their growth rate^18^ and to induce tolerance to salinity and temperature^40,41^. Here, exogenous NAA had a small, but not significant, effect in the light and reduced the extent to which the algae retreated from a high-light source. However, this effect was not substantial and there was no significant effect in the dark. In plants phototropism in response to blue light is regulated by the bacterial two component sensor-like, plasma membrane proteins, Phototropin 1 and 2 (Phot1 and Phot2)^42^, which interact with members of the NHP3 protein family, inducing their dephosphorylation^43^. The downstream signalling events are less clear, but the end result is the redistribution of auxin and differential growth between the shaded and illuminated sides of the plant organ. Phototropin also exists in Chlamydomonas and has been shown to be involved in the phototactic responses of this alga^44^. However, the NPH3 proteins appear to be specific to terrestrial plants and there are no obvious orthologues of the genes encoding these proteins in the Chlamydomonas genome. Indeed, the majority of the auxin signalling components of terrestrial plants appear to be absent in this alga^5^. Thus, this study would currently suggest that the phototactic responses of Chlamydomonas that occur *via* phototropin signalling and the gravitaxis response of the alga to ABA in the dark are not linked to the activity of auxin. It will, therefore, be important to identify the downstream protein targets of this photoreceptor in Chlamydomonas and to demonstrate how their activity is modulated by ABA.

Similarly, green algae are known to synthesise ACC and make ethylene^45^ and possess many recognisable components of the ethylene signal transduction pathway found in terrestrial plants^5,46^. Ethylene can be antagonistic to ABA responses in plants, *e.g*. during seed germination^47^ and shoot growth^48,49^. However, here, neither was there a significant effect of the exogenous application of ACC on the vertical positioning of the algae in the water column, nor any significant antagonistic effect on the observed ABA response in either the light or dark. Signalling *via* the second messenger, H_2_O_2_, has long been known to be required for the proper development of some plant ABA responses. For example, H_2_O_2_ synthesis by NADPH oxidases and its signalling are required for the ABA-induced guard cell movements that result in stomatal closure and its exogenous application also results in reduced stomatal apertures^50,51^. Here, such exogenous application of H_2_O_2_ did not mimic the effects of ABA and there was no significant effect on the position of the algae in the water column in either the light or the dark. Thus, it may be that many of the interactive ABA signalling processes that occur with the known phytohormones have, to a greater extent, evolved more recently in terrestrial plants.

These observations potentially provide insight into the early evolution of land plants and the ancient role of ABA in their development. Motility may have offered a survival advantage as algae evolved the protective biochemistry required for their transition to a terrestrial environment. During the diurnal variation of light intensity, motile algae may have moved up and down at the shoreline using light and ABA signalling to adjust their depth. As they adapted to high-light and desiccation, their ability to remain at the surface likely increased enabling them to become sessile and fixed in their light orientation. Such cells probably retained their core ABA signalling mechanisms while expanding downstream responses and interactions with other hormone signalling pathways as they developed multicellularity. Interestingly, the etiolation/de-etiolation response in plants appears to involve the expression of many ABA-related genes^52^ and plants grow upwards more in the pre-dawn hours than during the day^53^, suggesting retention of the algal ABA response described here. Thus, the results presented here suggest that ABA may be directly involved in plant tropisms that underpin such directional growth in the dark. Evidence that plant orientation results as much from phototropism^54^ and hydrotropism^55^ as from gravitropism is increasing and experiments conducted in micro-gravity have helped separate the contributions made by these tropisms in determining plant orientation and morphology^56^. ABA signalling may modulate plant phototropic and hydrotropic responses and future studies may further elucidate the interactive algal ABA and thus, terrestrial plant, molecular signalling mechanisms that operate to optimise their biochemistry in a changing environment.

## Materials and Methods

### Culturing of Chlamydomonas reinhardtii

All strains of *C. reinhardtii* were obtained from the Chlamydomonas Resource Centre, University of Minnesota, USA. Wild type *C. reinhardtii*^57^ strain CC-1021 mt+ and mutant strains CC-477 *bld1-1* and CC-2492 *pf13A* mt+ that lacked functional flagella were aseptically maintained at 23 °C under a 16 h photoperiod (45 µmoles photons m^−2^ s^−1^) on 1% (w/v) agar plates containing Tris-acetate/phosphate (TAP) media^58^ containing Hutner’s trace elements^59^. Final composition of the TAP media was 20 mM Tris-Acetate pH 7.0, 0.62 mM K_2_HPO_4_, 0.41 mM KH_2_PO_4_, 7.5 mM NH_4_Cl, 0.41 mM MgSO_4_, 0.34 mM CaCl_2_, 1.3 mM EDTA-disodium salt, 77 µM ZnSO_4_, 0.18 mM H_3_BO_3_, 26 µM MnCl_2_, 6.8 µM CoCl_2_, 6.3 µM CuSO_4_, 0.89 µM (NH_4_)_6_Mo_7_O_24_, 18 µM FeSO_4_, 0.3 mM KOH.

For liquid culture *C. reinhardtii* were inoculated from agar plates and were similarly grown in TAP media with rotational shaking (130 rpm) at 23 °C under a 16 h photoperiod (45 µmoles photons m^−2^ s^−1^). Growth of the algae in liquid cultures was monitored spectrophotometrically by following the δA_750_. The presence of predominantly motile zoospores and cell counts relating to A_750_ measurements were established microscopically and using a haemocytometer respectively.

### Effect of abscisic acid (ABA) on HCO_3_^−^ uptake by *C. reinhardtii* under different light levels

A 1.0 L culture of *C. reinhardtii* strain CC-1021 mt+ was grown until the mid-log phase (A_750_ = 0.3) and the cells were collected by centrifugation (200 × g, 5 min, 23 °C). The cell pellet was re-suspended in 50 mL of TAP media before mixing with 50 mL of 3% (w/v) sodium alginate and the suspension was subdivided before the addition of (+)ABA (Sigma, analytical reagent grade) to final concentrations of between zero and 50 µM. The alginate algal cell suspensions were immediately dropped through the nozzles of individual 25 mL syringes from a height of 40 cm into an excess volume of 0.18 M CaCl_2_ to form spherical 2 mm diameter gel beads of encapsulated algae either with or without ABA. Replicate (n = 5 *per* combination of ABA concentration and light level) 1 g aliquots of beads were then placed in sealed thin walled glass vials containing 19 mL of TAP media with 1 mL of freshly prepared bicarbonate indicator buffer (0.47 mM thymol blue, 0.27 mM cresol red, 100 mM NaHCO_3_, 1.1 M KCl) with no head space and were immediately placed in a light tunnel at distances from a photosynthetic light source (minus UV) that exposed the samples to a range of light intensities between 7.6 and 223.2 µmoles photons m^−2^ s^−1^. Samples were incubated at 23 °C for 1 h and the δA_550_ of the indicator buffer relative to time zero were recorded as an indication of the relative HCO_3_^−^ depletion of the media. The δA_550_ values were compared to a standard calibration to determine the changes in the concentration of HCO_3_^−^ in the TAP media effected by the algae within each of the treatment vials.

### Effect of phytohormones on the positioning of *C. reinhardtii* in the water column

Mid-log phase cultures of algae (A_750_ = 0.3), grown with a 16 h photoperiod (45 µmoles photons m^−2^ s^−1^) at 23 °C, were sampled at various time points (1, 8, 15, 20 and 23 h) during a 24 h cycle with the beginning of the photoperiod starting at time zero. Fully dispersed aliquots of the culture were transferred to 25 mL measuring cylinders to form 10 cm deep columns of algal cell suspensions. For phytohormone treatments, either (+) ABA from a 100 mM stock in 100% (v/v) ethanol, 1-naphthaleneacetic acid (NAA) from a 5.4 mM stock in 10 mM KOH, 1-aminocyclopropane-1-carboxylic acid from a 100 mM stock in water or hydrogen peroxide (H_2_O_2_) from a 100 mM stock in water were added to a final concentration of 50 µM. For control treatments an equivalent amount of stock solvent minus the phytohormone was added in each case. The cylinders were then either illuminated from above with high light (319.8 µmoles photons m^−2^ s^−1^) or were placed in the dark for 50 min at 23 °C. After incubation the positions of the algae were recorded photographically and the A_750_ of the cultures immediately measured at various depths from the surface of the water columns.

### Taxis of *C. reinhardtii* in response to a fixed ABA source in the light and dark

Mid-log phase cultures of the algae (A_750_ = 0.3), grown with a 16 h photoperiod, were sampled 8 h into the photoperiod and replicate (n = 5) fully dispersed 25 mL aliquots were dispensed into individual rectangular (1.5 × 1.5 × 12 cm) open-topped, light-permeable, acrylic troughs into which had been placed, at one end, agarose plugs containing various concentrations of (+) ABA between zero and 1 mM. The troughs were then immediately placed horizontally either in surrounding illumination (45 µmoles photons m^−2^ s^−1^) or in the dark and were incubated at 23 °C for 1 h. After incubation the positions of the algae in the tubes were recorded photographically and 200 µL samples simultaneously taken at incrementally increasing distances from the agarose plugs and their A_750_ measured.

### Measurements of endogenous ABA in *C. reinhardtii*

Replicate (n = 3) samples of known A_750_ and thus, known cell density, were taken at hourly intervals from a mid-log phase culture of *C. reinhardtii* grown under a 16 h photoperiod (45 µmoles photons m^−2^ s^−1^) at 23 °C. Aliquots (25 mL) of the culture were centrifuged to collect the algal cells and pellets were then immediately frozen in liquid N_2_ and freeze-dried. The freeze-dried pellets were then extracted and ABA contents determined using a competitive enzyme-linked immunosorbent assay^60^ kit (MyBioSource Inc., San Diego, CA, USA) as *per* the manufacturer’s instructions. Freeze dried algal pellets were extracted with 200 µL of kit sample extraction buffer for 16 h at 4 °C with rotational shaking at 60 rpm in the dark. This ratio of freeze dried algal cells to sample extraction buffer volume ensured that the final amounts of ABA measured in the test samples fell within the optimal detection range of the ELISA assay. Sample extracts were subsequently centrifuged (13,000 × g, 15 min 4 °C) and 50 µL aliquots of the clear supernatants were applied to individual wells of the ABA-coated ELISA plates. Aliquots (50 µL) of (+) ABA standards (0 to 10 µg mL^−1^) were similarly applied to adjacent wells. Next, 50 µL of anti-ABA antibody solution was added to each well, mixed and incubated at 37 °C for 30 min. Wells of the ELISA plate were subsequently aspirated and washed 3 times before the addition of 100 µL of horseradish peroxidase (HRP) second antibody conjugate and further incubation at 37 °C for 30 min. The ELISA plate wells were again aspirated and washed 5 times before the addition of 90 µL of TMB chromogenic substrate with incubation in the dark for 20 min at 37 °C. HRP activity was halted by the addition of 50 µL of stop solution and the fluorescence of individual wells of the ELISA plate was then measured at 450 nM using a FLUOstar Omega microplate reader (BMG Labtech). The amount of ABA in the test samples was then determined by comparison of the sample 450 nm readings with the curve generated from those of the standards applied to the ELISA plate. The amounts of ABA determined in each test sample were then divided by the number of algal cells extracted in each case and the results expressed as µg ABA algal cell^−1^.

### Data handling and statistical analysis

Where appropriate, groups of data were assessed for normality by Shapiro Wilk tests and for between group homogeneity of variances around the means by Bartlett’s tests. For HCO_3_^−^ depletion data, overall between data group comparisons of means were made by one and two way ANOVAs and *post hoc* pairwise mean comparisons by Tukey Kramer tests. For the algal movement data, comparisons of the relative distributions of the algae in response to the treatments were undertaken using χ^2^ tests of independence with *post hoc* χ^2^ tests of goodness of fit. Endogenous ABA data was analysed by one way ANOVA and selected pairwise comparisons by 2 sample t-tests. All tests were performed at α = 0.05 level of confidence.

## Data Availability

The data generated during and/or analysed during the current study are available from the corresponding author on reasonable request.
